# Epitelial-to-mesenchimal transition and invasion are upmodulated by tumor-expressed granzyme B and inhibited by docosahexaenoic acid in human colorectal cancer cells

**DOI:** 10.1186/s13046-016-0302-6

**Published:** 2016-02-02

**Authors:** Donatella D’Eliseo, Giuliana Di Rocco, Rossella Loria, Silvia Soddu, Angela Santoni, Francesca Velotti

**Affiliations:** Department of Molecular Medicine, Istituto Pasteur-Fondazione Cenci Bolognetti, Sapienza University of Rome, 00161 Rome, Italy; Department of Ecological and Biological Sciences (DEB), La Tuscia University, Largo dell’Università, 01100 Viterbo, Italy; Department of Research, Advanced Diagnostics, and Technological Innovation, Regina Elena National Cancer Institute, 00144 Rome, Italy

**Keywords:** Granzyme B, Epithelial-to-mesenchymal transition, Invasion, Colorectal cancer, Cancer stem cells, Transforming growth factor-β, Docosahexaenoic acid, ω-3 Polyunsaturated fatty acid, Adjuvant cancer therapy

## Abstract

**Background:**

Granzyme B (GrB) is a serine protease, traditionally known as expressed by cytotoxic lymphocytes to induce target cell apoptosis. However, it is emerging that GrB, being also produced by a variety of normal and neoplastic cells and potentially acting on multiple targets, might represent a powerful regulator of a wide range of fundamental biological processes. We have previously shown that GrB is expressed in urothelial carcinoma tissues and its expression is associated to both pathological tumor spreading and EMT. We have also shown that docosahexaenoic acid (DHA), a dietary ω-3 polyunsaturated fatty acid with anti-tumor activity, while inhibiting urothelial and pancreatic carcinoma cell invasion also inhibited their GrB expression in vitro. In this study, we characterized a panel of colorectal carcinoma (CRC) cells, with different invasive capabilities, for GrB expression and for the contribution of GrB to their EMT and invasive phenotype. In addition, we investigated the effect of DHA on CRC cell-associated GrB expression, EMT and invasion.

**Methods:**

The expression levels of GrB and EMT-related markers were evaluated by Western blotting. GrB knockdown was performed by Stealth RNAi small interfering RNA silencing and ectopic GrB expression by transfection of human GrB vector. Cell invasion was determined by the BioCoat Matrigel invasion chamber test.

**Results:**

GrB was produced in 57.1 % CRC cell lines and 100 % CRC-derived Cancer Stem Cells. Although GrB was constitutive expressed in both invasive and noninvasive CRC cells, GrB depletion in invasive CRC cells downmodulated their invasion in vitro, suggesting a contribution of GrB to CRC invasiveness. GrB loss or gain of function downmodulated or upmodulated EMT, respectively, according to the analysis of cancer cell expression of three EMT biomarkers (Snail1, E-cadherin, N-cadherin). Moreover, TGF-β1-driven EMT was associated to the enhancement of GrB expression in CRC cell lines, and GrB depletion led to downmodulation of TGF-β1-driven EMT. In addition, DHA inhibited GrB expression, EMT and invasion in CRC cells in vitro.

**Conclusions:**

These findings present a novel role for GrB as upmodulator of EMT in CRC cells. Moreover, these results support the use of DHA, a dietary compound without toxic effects, as adjuvant in CRC therapy.

## Background

Colorectal cancer (CRC) represents the second most common cause of cancer-related death in the Western world [1]. Although treatment outcomes in patients with CRC have improved over the past two decades, 5-year relative survival is slightly greater than 10 % in patients with metastatic CRC, being metastasis and drug resistance the primary cause of mortality and thus the most life-treating aspects [1].

Epithelial-to-mesenchymal transition (EMT) has been linked to the initiation of metastasis and to drug resistance across multiple organs, including CRC [2, 3]. A reciprocal relationship exists between EMT and Cancer Stem Cells (CSCs), in that CSCs often exhibit EMT and EMT can lead to the formation of CSCs [1, 2, 4]. EMT, consisting in the transdifferentiation of epithelial cells into mesenchymal cells, is physiological in embryonic development, where it takes place as a coordinated and organized process, leading epithelial cells to acquire the motile and invasive characteristics of mesenchymal cells. Potent initiators of EMT are some growth factors, including transforming growth factor (TGF)-β, whose activated receptors trigger intracellular signaling cascades ultimately resulting in the downregulation of epithelial (E)-cadherin, a major hallmark of EMT, often balanced by the increased expression of mesenchymal cell markers such as neural (N)-cadherin [2]. The zinc finger molecule SNAI1/Snail 1 is one of the critical E-cadherin transcriptional repressors in EMT [2, 5]. Unlike developmental EMT, the molecular network in tumor-associated EMT is disregulated and EMT-activated carcinoma cells, depending on tissue, signaling and their context, may lose their epithelial characteristics partially (incomplete EMT) or completely (complete EMT), covering a range of changes in their differentiation markers as well as in their invasive migratory behavior [2, 6–8]. The molecular processes underlying EMT and linked to cancer progression are complex and still partially understood. Therefore, the advancing knowledge on molecules involved in tumor-associated EMT is essential to better understand the mechanisms underling tumor metastasis and drug resistance, as well as to improve cancer therapies.

Granzyme B (GrB) is a serine protease traditionally known as molecule expressed by cytotoxic lymphocytes to cause apoptosis in tumor and virally infected cells, primarily by activation of caspase-driven pathways [9, 10]. However, it is now recognized that GrB is also produced by other non-cytotoxic immune and nonimmune cells as well as neoplastic cells [11–19], and that consensus sites for GrB cleavage are present in several intra- and extra-cellular proteins [20, 21]. They include intracellular proteins involved in cell anchorage, signaling, and cycle regulation, as well as extracellular matrix (ECM) components, cell-surface receptors, pro-inflammatory cytokines and growth factors [12, 21–23]. Consequently, GrB is emerging as a multifunctional protease, acting with context dependence and potentially playing important roles in different diseases, including inflammatory and degenerative diseases [24].

We have previously demonstrated that GrB is expressed in urothelial carcinoma tissues and its expression is associated to both pathological tumor spreading and EMT [18]. We have also shown that GrB expressed in bladder and pancreatic carcinoma cells promotes their invasion in vitro [18, 25]. Finally, we have observed that docosahexaenoic acid (DHA; 22:6), a long-chain ω-3 polyunsaturated fatty acid primarily found in dietary fish oil [26–29], inhibits tumor-associated GrB expression in bladder and pancreatic carcinoma cells while simultaneously suppresses their invasion in vitro, suggesting the involvement of GrB in the inhibition of tumor cell invasion by DHA [25]. A large number of experimental and preclinical studies have shown that DHA exerts selective anticancer activity and inhibits the development and the progression of some types of cancers, including CRCs [26–31]. Currently, clinical trials underline the potential value of DHA as an effective adjuvant to cancer therapy [28, 32–35]. However, the mechanisms underlying the anticancer activity of DHA remain unclear. Taking into account the above mentioned data, in this study we report the characterization of a panel of CRC cells, with different invasive capabilities, for GrB expression and for the contribution of GrB to their EMT and invasive phenotype. In addition, the effect of DHA on GrB expression, EMT and invasion in CRC cells was analyzed in vitro.

## Methods

### Cells and cultures

Seven human established CRC cell lines (HT-29, SW480, SW620, Caco-2, LoVo, HCT 116, HCT-8), two human bladder (RT112, T24) and one pancreatic (PT45) cancer cell lines were purchased from American Type Culture Collection (ATCC, Manassas, VA, USA) or kindly provided by collaborators. Four CRC patient-derived CSCs were kindly provided by Prof. R. De Maria and G. Stassi [36, 37]. YT-S human cytotoxic NK leukemia cells and T24 human bladder cancer cells served as positive and negative controls for GrB expression, respectively. All cell lines were grown in RPMI-1640 (Sigma-Aldrich, St. Louis, MO, USA), but HT-29, SW480, Caco-2 and HCT 116 in DMEM (Sigma-Aldrich), supplemented with 10 % FCS (Hyclone, South Logan, UT, USA), 100 mg/ml streptomycin and 100 IU/ml penicillin (EuroClone S.p.A., MI, Italy), 25 mM HEPES and 2 mM L-glutamine (EuroClone S.p.A.). CSCs were grown as spheroids in non-adherent conditions, as previously described [36]. To induce EMT, cells were incubated with 10 ng/ml TGF-β1 (PeproTech EC Ltd, London, UK). In some experiments, cells were pretreated with 30 μM brefeldin A (Sigma-Aldrich), and 4 mL of supernatants were collected and concentrated to approximately 0.15 mL, using a Centricon Amicon® Ultra-4 centrifugal filter unit (Merck Millipore, Darmstadt, Germany). In other experiments, cells were treated with 100 μM DHA (Sigma-Aldrich) dissolved in ethanol solution or with ethanol solution alone for 24 h.

### Reverse transcription polymerase chain reaction (RT-PCR)

Total RNA was isolated using the RNeasy mini Kit (Qiagen). cDNA was synthesized from using a M-MLTV RTase and amplified with GoTaq DNA polymerase (Promega). For the detection of Primer sequences are as follows: Lgr5-FW: CCCGAATCCCCTGCCCAGTCT; Lgr5-REV: TCATCCAGCCACAGGTGCCTCA; Bmi1-FW: CGCGCTGGTTGCCCATTGAC; Bmi1-REV: AGCACACACATCAGGTGGGGA; CD133-FW: GCAGCAGTCTGACCAGCGTGA; CD133-REV: GCCGCACACGCCACACAGTA; CD166-FW: CCCCTTGAAGGAGCGGTGGTC; CD166-REV: CCTCTGGGGGAGGGTTGCCAT; GAPDH-FW: AGCCTCCCGCTTCGCTCTCT; GAPDH-REV: GCCAGCATCGCCCCACTTGA.

### GrB knockdown by small interfering RNA (siRNA)

Three 25-mer Stealth RNAi duplexes targeting human GrB and Control Stealth RNAi were obtained by the BLOCK-iT RNAi design program (Invitrogen, Carlsbad, CA, USA). For Stealth RNAi duplexes 1: 5′-CCU ACA UGG CUU AUC UUA UGA UCU G-3′ and 5′-CAG AUC AUA AGA UAA GCC AUG UAG G-3′; for Stealth RNAi duplexes 2: 5′-GCG AAU CUG ACU UAC GCC AUU AUU A-3′ and 5′-UAA UAA UGG CGU AAG UCA GAU UCG C-3′ and for Stealth RNAi duplexes 3: 5′-GCC UGC ACC AAA GUC UCA AGC UUU G-3′ and 5′-CAA AGC UUG AGA CUU UGG UGC AGG C-3′. A pool of 3 different nonsilencing, nonoverlapping RNAi duplexes with matched GC content, served as Control Stealth RNAi. Transfection was performed using INTERFERin™ reagent (Polyplus transfection SA, Illkirch, France). In addition, for siGrB#2, three 27-mer siRNA duplexes targeting human GrB (SR302035) and Universal scrambles negative control siRNA duplex (SR30004) were obtained by OriGene (OriGene Technologies, Rockville, MD, USA). For siRNA duplexes 1# (SR302035A): 5′-GGA AGA UCG AAA GUG CGA AUC UGA C-3′; for siRNA duplexes 2# (SR302035 B): 5′-GCA ACU GAA UAA AUA CCU CUU AGC T-3′; for siRNA duplexes 3# (SR302035C): 5′-ACC AUG AAA CGC UAC UAA CUA CAG G-3′.

### GrB plasmid transfection

Vector for human GrB (Homo sapiens GranzymeB cDNA Clone, Sino Biological Inc, Beijing, China) and Mock were transfected into 70–80 % confluent cells in Opti-MEM (Gibco, Invitrogen), using Lipofectamine LTX and PLUS reagents (Invitrogen).

### Western blotting

Cell lysates were prepared by a solution containing 50 mM Tris–HCl pH 7.6, 150 mM NaCl, 0.5 % TRITON X-100, 0.5 % Sodium deoxycolate, 0.1 % SDS and the protease inhibitor mixture “Complete” (Roche Diagnostic GmbH, Germany). Proteins (30–80 μg for CRC cells; 7.5 μg for YT-S cells) were separated by SDS-PAGE, blotted onto nitrocellulose (Whatman-Protan BA85, GE Healthcare, NJ, USA) and incubated with appropriated primary antibodies specific for: GrB (2C5/F5; Chemicon, Prodotti Gianni, Italy), E-cadherin (36; BD Transduction Laboratories, CA, USA), N-cadherin (3B9; Zymed, Invitrogen), SnaI1 (C15D3; Cell Signaling Technology, MA, USA) or β-actin Ac-40 (Sigma-Aldrich). The reaction was revealed by horseradish peroxidase (HRP)-coupled secondary reagents (Bio-Rad, Hercules, CA, USA) and developed by enhanced chemiluminescence (Amersham ECL Western Blotting Detection Reagent, GE Healthcare, MI, Italy). Band intensities (b.i.) = band volume/area x mean pixel intensity, normalized for β-actin, were quantified using Quantity One 1-D analysis software (Bio-Rad).

### Cell growth and viability assessment

Cell growth was assessed determining, in triplicate, the cell number with a Neubauer cell counter (Brand West Germany). Cell viability was assessed by the trypan blue dye exclusion assay.

### Invasion assay

Cells, in serum free medium, were added in triplicate to the upper well in BioCoat Matrigel Invasion Chambers (Corning Inc, Corning, NY, USA), allowed to adhere for 2 h and to migrate toward 10 % FCS for 24–48 h (cell line-dependent time) at 37 °C. Cells underside of the membrane were fixed, stained with a solution containing 50 % isopropanol, 1 % formic acid and 0.5 % (wt/vol) brilliant blue R 250 (Bio-Rad), and counted using a light microscope. Results are presented by mean *±* standard deviation (S.D.) of triplicates.

### Statistical analysis

Student’s *t* test was used for all analyses; *p* < 0.05 was considered significant.

## Results

### GrB expression in CRC cells and their invasive phenotype

**Fig. 1:**
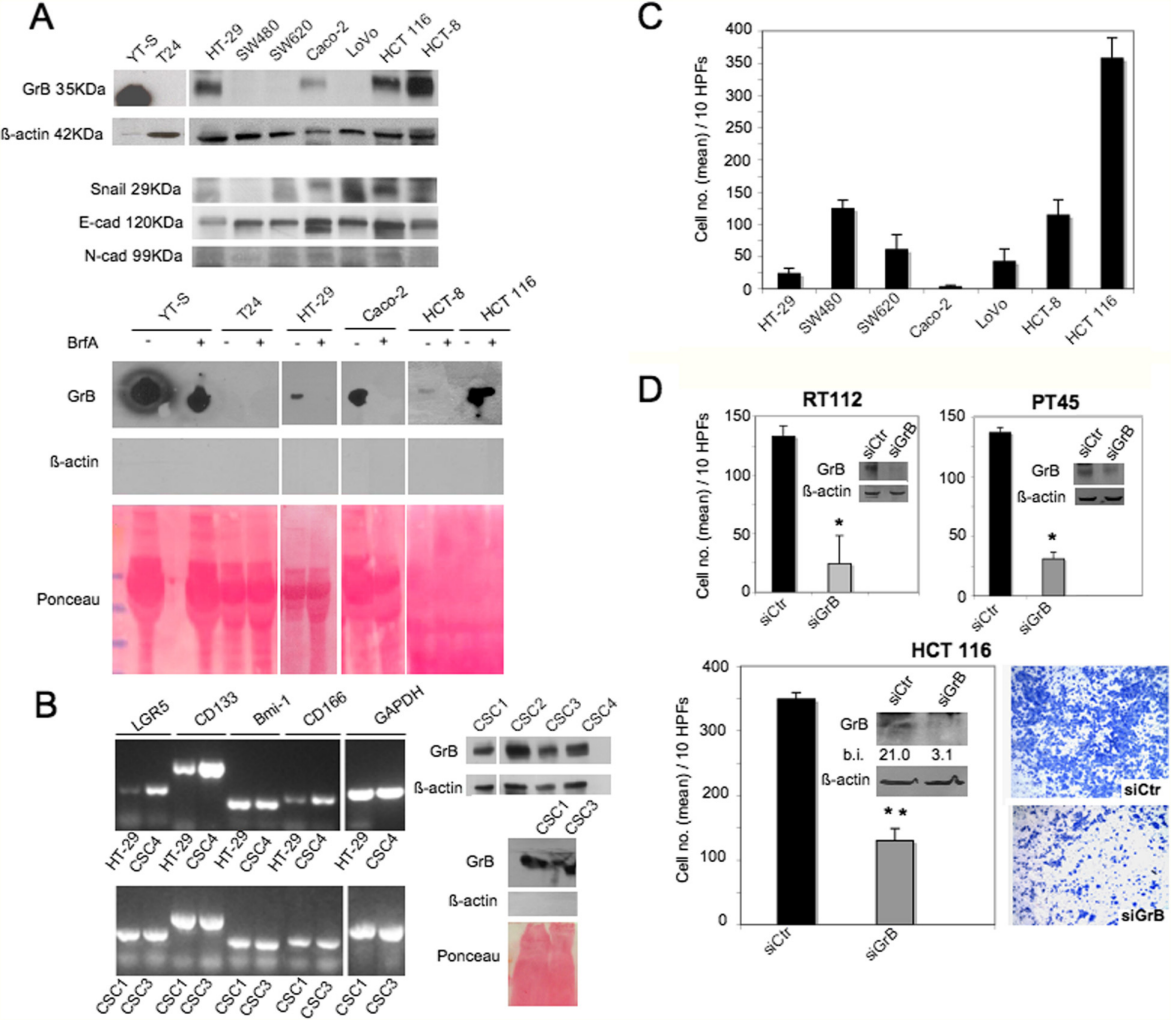
GrB production and invasion in CRC cells. **a** (*upper panel*) GrB expression and EMT biomarkers (Snail 1, and E- and N-cadherin) were analyzed in CRC cell lines by WB on cell lysates; β-actin was used as loading control; **a** (*lower panel*) GrB secretion was investigated in the indicated cells pretreated with (+) or without (−) brefeldin A (BrfA), their supernatants were collected and analyzed by WB; β-actin was used as intracellular protein control and Ponceau staining as loading control. YT-S (NK leukemia) and T24 (bladder cancer) cells were used as positive and negative controls, respectively. **b** (*left panel*) The expression of colon stem cell markers such as Lgr5, Cd133 and CD166 and Bmi1 were analyzed in CSCs *vs* HT-29 cell lines by RT-PCR; GAPDH was used as loading control; **b** (*right panel*) GrB expression and secretion were analyzed in the indicated CSCs as in (**a**); **c** Invasion of CRC cells using the BioCoat Matrigel Invasion Chamber test; **d** HCT 116 cells were transfected with GrB-specific Stealth RNAi (siGrB) or Control Stealth RNAi (siCtr); GrB expression was verified by WB; β-actin was used as loading control; numbers indicate band intensities (b.i.) = band volume/area x mean pixel intensity, normalized for β-actin and quantified using Quantity One 1-D analysis software; invasion assay was performed using the BioCoat Matrigel Invasion Chambers test; bladder RT112 and pancreatic PT45 cancer cells served as positive controls; * *p* < 0.0001; **p < 0.001. Representative experiments out of at least three

We investigated GrB production in CRC cells and its role in their invasive capability. GrB expression was analyzed in a panel of 7 established CRC cell lines and in 4 CRC patient-derived CSCs [36, 37]. A band at ∼ 35 kDa corresponding to GrB was found by Western blotting (WB) in 4 (HT-29, Caco-2, HCT 116 and HCT-8) out of 7 (57.1 %) CRC cells (Fig. 1a, upper panel) and in 4 out of 4 (100 %) CSCs (Fig. 1b, right panel). As previously found for bladder carcinoma cells [18], different band intensities appeared in different CRC cells and they were always much weaker than that in YT-S cells (the NK leukemia cell line used as positive control), indicating that GrB levels vary among cancer cells and they are always significantly lower than that in cytotoxic lymphocytes. We also examined whether tumor-expressed GrB was released into the extracellular milieu. Conditioned media from GrB positive cell cultures were collected and GrB measured by WB. As shown in Fig. 1a (lower panel) and 1b (right panel), GrB was released by GrB positive cells. Moreover, pre-treatment of CRC cells with 30 μM brefeldin A, an inhibitor of protein transport from endoplasmic reticulum to Golgi apparatus, prevented GrB release, indicating the secretion of GrB by CRC cells (Fig. 1a, lower panel).

Next, to investigate the function of GrB in CRC cell invasion, we first characterized CRC cell lines for both their expression of three EMT biomarkers (i.e., Snail 1 transcription factor and E- and N-cadherin adhesion molecules) by WB (Fig. 1a, upper panel) and their invasive capability by the invasion assay (Fig. 1c). Consistent with the literature [38–41], SW480, HCT-8 and HCT 116 cells resulted the most invasive cells, while HT-29, Caco-2, SW620 and LoVo cells exhibited low or no invasiveness (Fig. 1c). Then, to investigate GrB function in CRC cell invasion, we selected HCT 116 out of the panel of CRC cells, because of both its GrB expression and its high invasiveness. According to the results obtained in RT112 bladder and PT45 pancreatic cancer cells [18, 25], GrB knockdown in HCT 116 cells significantly inhibited their invasion (Fig. 1d), indicating that GrB can also promote invasion in CRC cells. In addition, as illustrated in Fig. 1a and c*,* comparable levels of GrB constitutive expression were present in both invasive (HCT-8 and HCT 116) and very lowly invasive (HT-29 and CaCo-2) cells, suggesting that other factors linked to the cell context might interfere with the promotion of invasion by GrB.

### GrB upmodulates tumor-associated EMT

**Fig. 2:**
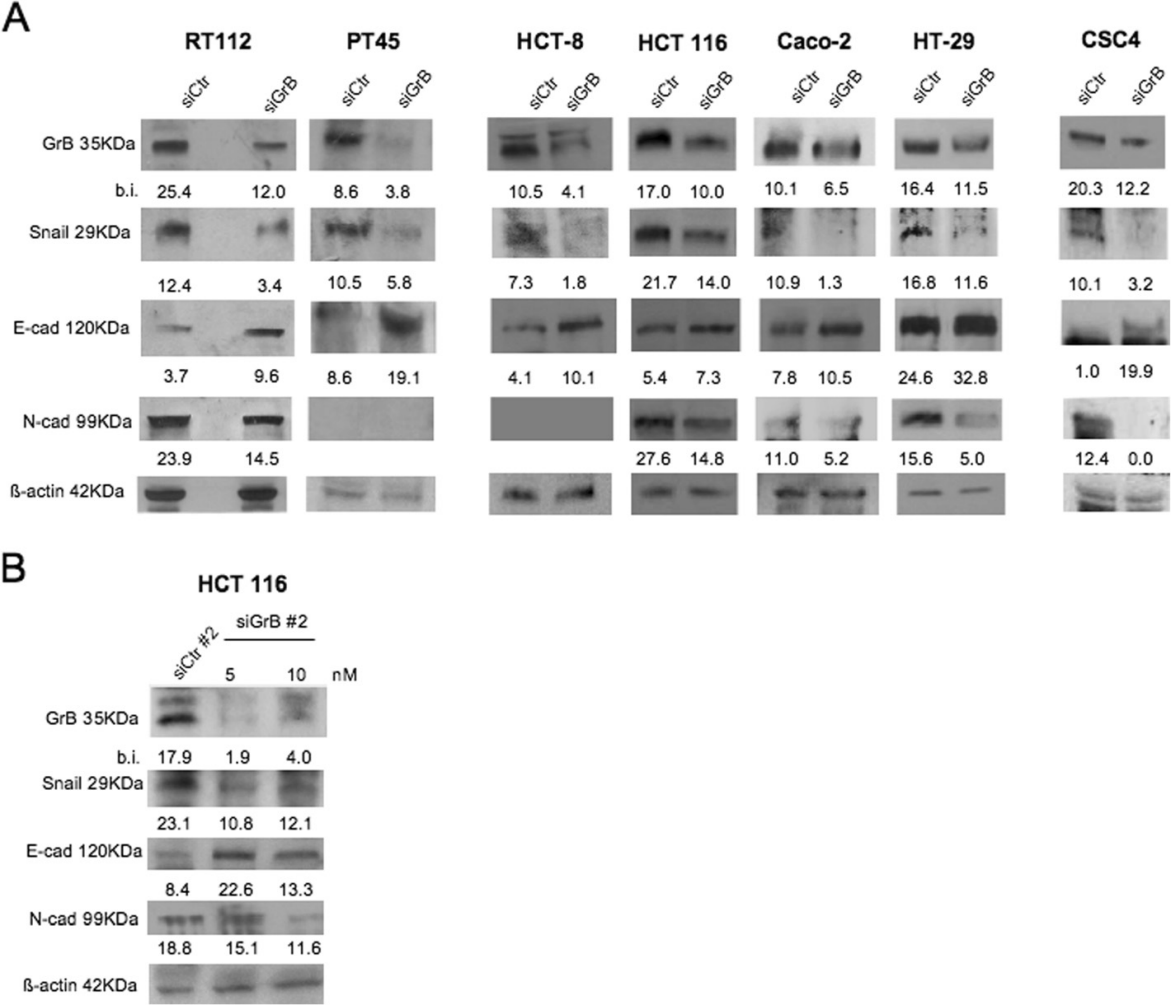
GrB depletion downmodulates EMT in cancer cells. The indicated GrB positive CRC cells and CSC4 were transfected with (**a**) GrB-specific Stealth RNAi (siGrB) or Control Stealth (siCtr) RNAi; GrB depletion was verified by WB; EMT was investigated analyzing the expression of 3 EMT biomarkers (Snail 1, and E- and N-cadherin) by WB; β-actin was used as loading control; numbers indicate band intensities (b.i.) = band volume/area x mean pixel intensity, normalized for β-actin and quantified using Quantity One 1-D analysis software; and (**b**) another GrB siRNA (siGrB#2) than in (**a**), targeting the same gene at different sequence; the experiment was performed as in (**a**). Representative experiments out of at least three

To investigate the functional relationship between tumor-expressed GrB and EMT, we knocked-down GrB in highly (HCT-8 and HCT 116) and lowly (Caco-2 and HT-29) invasive GrB positive CRC cell lines as well as in RT112 bladder and PT45 pancreatic cancer cells. The transfectable CSC4 was also included in the experiment. Then, we evaluated EMT by WB, analyzing the expression of the three EMT biomarkers (Snail 1, E-cadherin and N-cadherin). As shown in Fig. 2a, GrB depletion was associated to the increase of epithelial E-cadherin expression and the decrease of the mesenchymal markers Snail 1 and N-cadherin (when present) in all tumor cells, independently of their invasive capability, suggesting a contribution of GrB in EMT promotion. Moreover, to exclude siRNA non-specific effects, another GrB siRNA (siGrB#2), targeting the same gene at different sequence, was used to deplete GrB in HCT 116 cells. As shown in Fig. 2b, GrB depletion was associated to the increase of EMT biomarkers, confirming the result obtained in Fig. 2a.

**Fig. 3:**
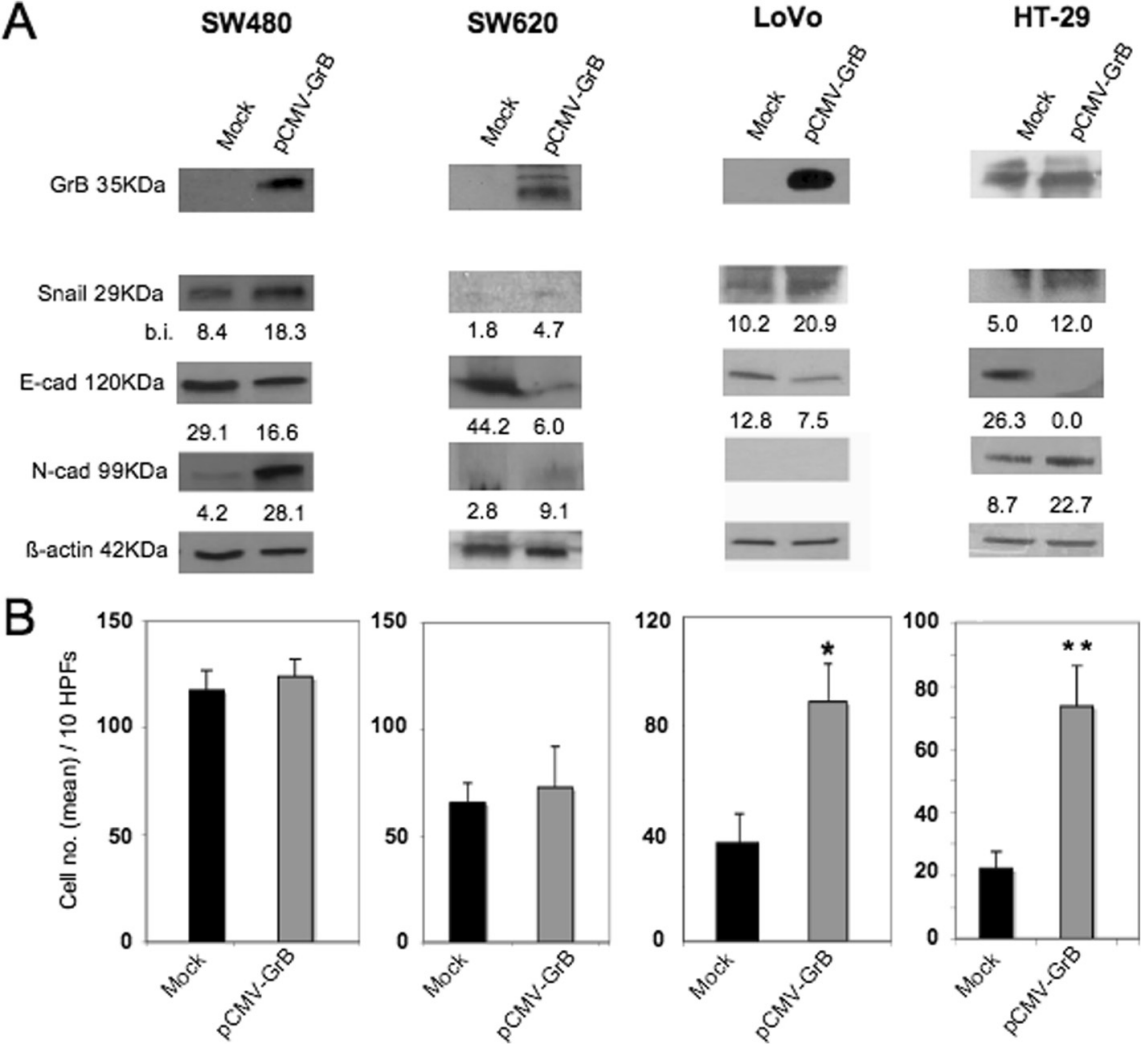
Ectopic GrB upmodulates EMT in CRC cells. GrB negative (SW480, SW620, LoVo) and GrB positive (HT-29) CRC cells were transfected with human GrB vector (pCMV-GrB); Mock-transfected cells were used as control. **a** GrB transfection efficacy was verified by WB; EMT was investigated analyzing the expression of 3 EMT biomarkers (Snail 1, and E- and N-cadherin) by WB; β-actin was used as loading control; numbers indicate band intensities (b.i.) = band volume/area x mean pixel intensity, normalized for β-actin and quantified using Quantity One 1-D analysis software. **b** Invasion assay was performed using cells transfected with GrB (pCMV-GrB) or Mock; * *p* < 0.01; **p < 0.0001. Representative experiments out of at least three

To further investigate GrB function in EMT, we examined whether GrB transfection in CRC cells affected their EMT phenotype. To this purpose, GrB negative (SW480, SW620 and LoVo) and positive (HT-29) CRC cells, with different invasive capabilities, were transfected with the human GrB vector and EMT biomarkers were evaluated by WB. As shown in Fig. 3a, ectopic GrB expression (compatible with the endogenous expression levels) upmodulated EMT in all CRC cells, driving epithelial tumor cells towards a mesenchymal phenotype. However, it might also be noted that N-cadherin was upmodulated in SW480 and HT-29 cell lines, both derived from primary tumors, but not in metastasis-derived SW620 and LoVo cells, which might not have the competence to undergo clear EMT since they have very likely passed through it before. In addition, we observed that upmodulation of EMT biomarkers by ectopic GrB was associated to the enhancement of invasion in HT-29 and LoVo cells, but not in SW480 and SW620 (Fig. 3b). This finding might further support both the hypothesis that other cell factors might interfere with the promotion of invasion by GrB and the data from the literature reporting that EMT-activated neoplastic cells may undergo incomplete EMT, not always coupled to the invasive cell migration behavior [2, 6–8]. Altogether, our results indicate that GrB can modulate the tumor EMT process.

### GrB contributes to the induction of EMT driven by TGF-β1 in CRC cells

**Fig. 4:**
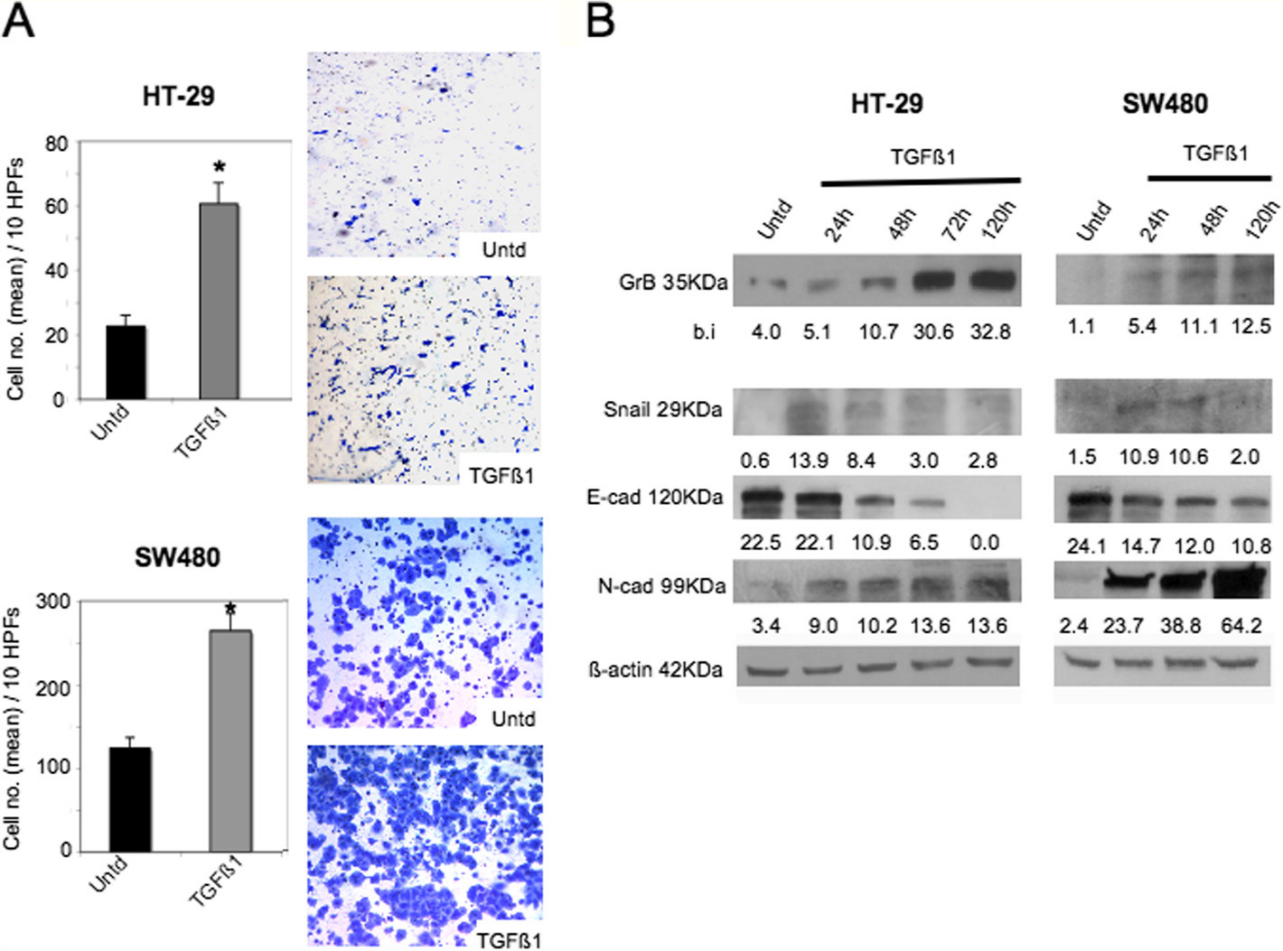
TGF-β1-driven EMT is associated to enhancement of GrB expression. CRC cells were induced to undergo EMT by their incubation with TGF-β1 different time points; cells treated (TGF-β1) or untreated (Untd) with TGF-β1 were analyzed for (**a**) invasion through Matrigel at 24 h (*p < 0.0001) and (**b**) GrB expression and 3 EMT biomarkers (Snail 1, and E- and N-cadherin) by WB; β-actin was used as loading control; numbers indicate band intensities (b.i.) = band volume/area x mean pixel intensity, normalized for β-actin and quantified using Quantity One 1-D analysis software. Representative experiments out of three

TGF- β1 signaling plays a key role in tumor progression via EMT in many tumors, including CRCs [2, 3]. To further investigate the functional relationship between GrB and EMT, CRC cells were induced to undergo EMT by TGF-β1 GrB expression was analyzed by WB. Among our CRC cell panel, it is known that HCT 116 and HCT-8 are unresponsive to TGF- β1 because of their lack of the TGF-β type II receptor, whereas HT-29 and SW480 are sensitive to TGF- β1 stimulation [42]. Thus, GrB positive HT-29 and GrB negative SW480 cells were chosen to be treated with TGF-β1 for different time periods and, as expected [42], at 48–72 h they underwent morphologic changes consistent with EMT (e.g., increased intercellular separation and spindle-cell shape) (data not shown) associated with ∼ 3 fold invasion increase (*p* < 0.0001) (Fig. 4a). Consistent with the literature [42]*,* WB analysis revealed the transition of carcinoma cells towards a mesenchymal phenotype, with the upregulation of Snail 1 expression at 24 h, associated with a time-dependent decrease of E-cadherin and increase of N-cadherin (Fig. 4b). Interestingly, in addition to the modulation EMT biomarkers, we observed a time-dependent accumulation of GrB expression in both cell lines (Fig. 4b)*.* In particular, we observed a significant increase of GrB levels in GrB positive HT-29 cells and the induction of GrB expression in the GrB negative SW480 cell line. These results might suggest the involvement of GrB in TGF-β1-mediated EMT.

**Fig. 5:**
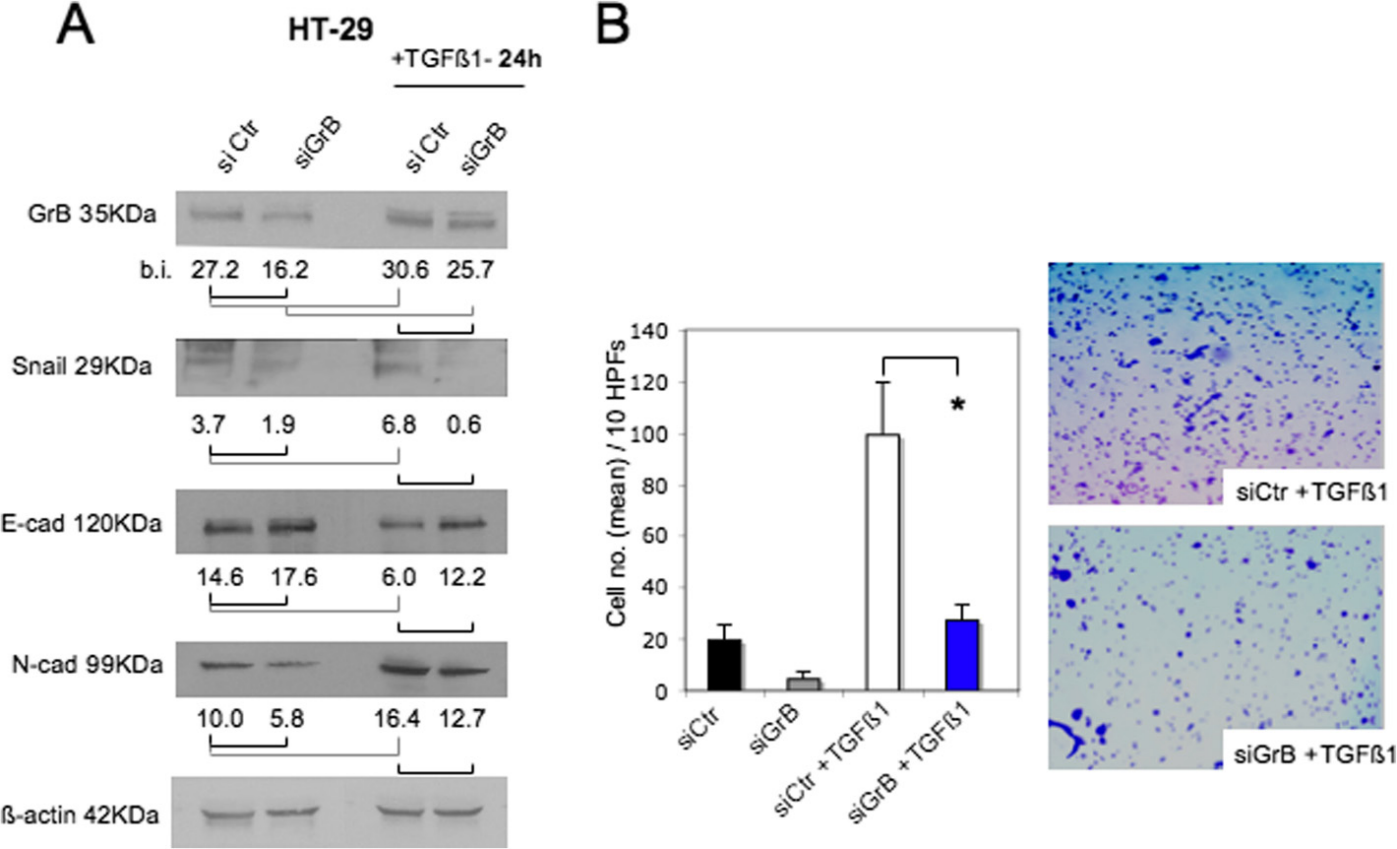
GrB depletion downmodulates TGF-β1-driven EMT in CRC cells. First, we knocked-down GrB in GrB positive HT-29 cells, then, we incubated cells with TGF-β1 and analyzed EMT and invasion. **a** GrB depletion was verified by WB in CRC cells treated or not, as indicated; EMT biomarkers (Snail 1, and E- and N-cadherin) were analyzed by WB; β-actin was used as loading control; numbers indicate band intensities (b.i.) = band volume/area x mean pixel intensity, normalized for β-actin and quantified using Quantity One 1-D analysis software. **b** HT-29 cell invasion through Matrigel was performed using cells transfected with GrB-specific (siGrB) or Control (siCtr) Stealth RNAi and then treated or not with TGF-β1; *p < 0.0001. Representative experiments out of three

To directly assess the causative contribution of GrB to the induction of EMT driven by TGF-β1, we first knocked-down GrB in the GrB positive HT-29 cell line, then we treated cells with TGF-β1 and analyzed EMT. We choose HT-29 cells, because, unlike SW480, in HT-29 cells GrB can modulate both EMT biomarkers and invasive behavior (Fig. 3). Efficient GrB silencing was confirmed by WB (Fig. 5a). As shown in Fig. 5a, GrB depletion downmodulated TGF-β1-induced EMT, as assessed by E-cadherin increase and Snail 1 and N-cadherin decrease, as well as cell invasion inhibition (Fig. 5b). Taken together, these results confirm the contribution of GrB to the induction of EMT by TGF-β1.

### DHA inhibits GrB expression, EMT and invasion in CRC cells

**Fig. 6:**
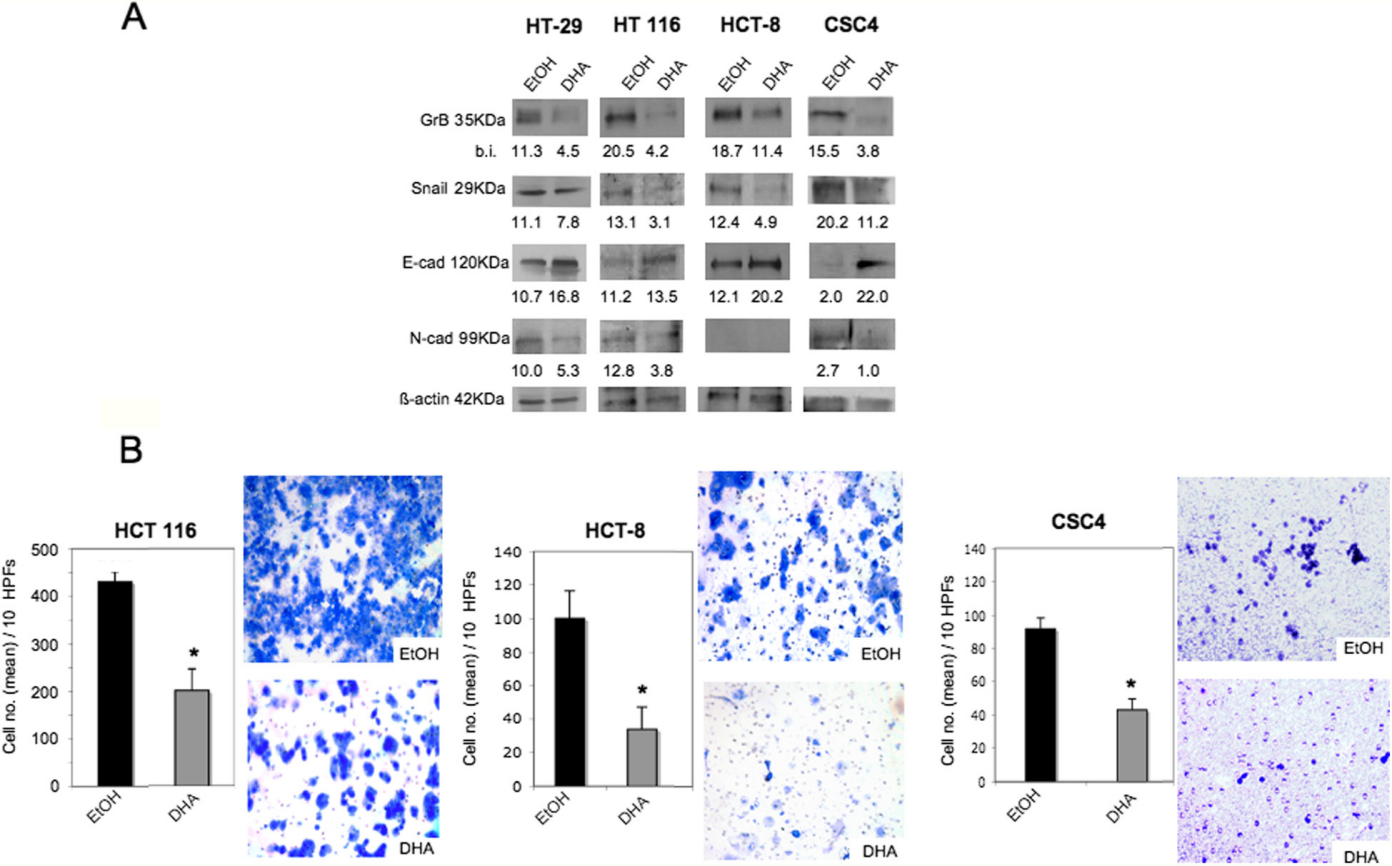
DHA inhibits GrB expression, EMT and invasion in CRC cells. GrB positive CRC cells were treated with 100 μM DHA dissolved in ethanol solution or with ethanol solution alone (EtOH) for 24 h, and (**a**) GrB expression and 3 EMT biomarkers (Snail 1, and E- and N-cadherin) were analyzed by WB; β-actin was used as loading control; numbers indicate band intensities (b.i.) = band volume/area x mean pixel intensity, normalized for β-actin and quantified using Quantity One 1-D analysis software; (**b**) invasion was assessed in the invasive HCT 116, HCT-8 cell lines and in CSC4; *p* < 0.0001. Representative experiments out of at least three

Finally, we investigated whether DHA, a dietary natural compound known for its selective anticancer properties [26–28], inhibited GrB expression as well as EMT and invasion in CRC cells. GrB positive HT-29, HCT-8, HT 116 cell lines and CSC4 were treated with 100 μM DHA for 24 h, and the expression of GrB and EMT biomarkers was analyzed by WB. This DHA concentration had no effects on cell proliferation, as verified by cell count and by the viability assay (data not shown). As shown in Fig. 6a, 100 μM DHA significantly inhibited GrB and modulated EMT biomarkers in all CRC cells tested. In addition, the same DHA dose inhibited invasion through Matrigel of the invasive HCT 116 and HCT-8 cell lines as well as of CSC4 (Fig. 6b).

## Discussion

It is emerging that the serine protease GrB, being produced by a variety of normal and neoplastic cells and potentially acting on multiple targets, might represent a powerful regulator of a wide range of fundamental biological processes [21–24, 43, 44]. Currently, GrB is widely used as activation marker for cytotoxic lymphocytes, and lymphocyte-derived GrB positive tumor immunostaining is associated with a favorable clinical outcome in a large spectrum of cancers [45]. However, the relative importance of GrB in in vivo cytotoxicity by CTLs has been questioned [46], and, in some cases, GrB expression at the tumor site correlates to the severity of the disease, poor prognosis and resistance to therapy [45, 47–51]. We have previously documented that GrB is expressed by urothelial carcinoma tissues and its expression is associated to increasing pathological tumor spreading as well as to EMT [18]. Significantly, GrB expression was concentrated in urothelial neoplastic cells undergoing EMT at the cancer invasion front [18].

Here, we showed the production and secretion of GrB in a panel of CRC cells and we presented a novel role for GrB as upmodulator of tumor-associated EMT. We documented the expression of GrB not only by 57.1 % of established CRC cell lines, but also, interestingly, by 4 out of 4 CRC patient-derived CSCs, which are known to play a pivotal role in tumor metastasis, tumor regeneration and drug resistance. We showed that GrB knockdown in CRC cells as well as in bladder and pancreatic carcinoma cells was sufficient to increase E-cadherin and decrease Snail and N-cadherin (when present) expression. Conversely, ectopic GrB expression led to the decrease of E-cadherin and the increase of Snail 1 expression, associated to the enhancement of N-cadherin in primary CRC cells. Altogether, these data indicate that GrB contributes to the activation of EMT in tumor cells.

**Fig. 7:**
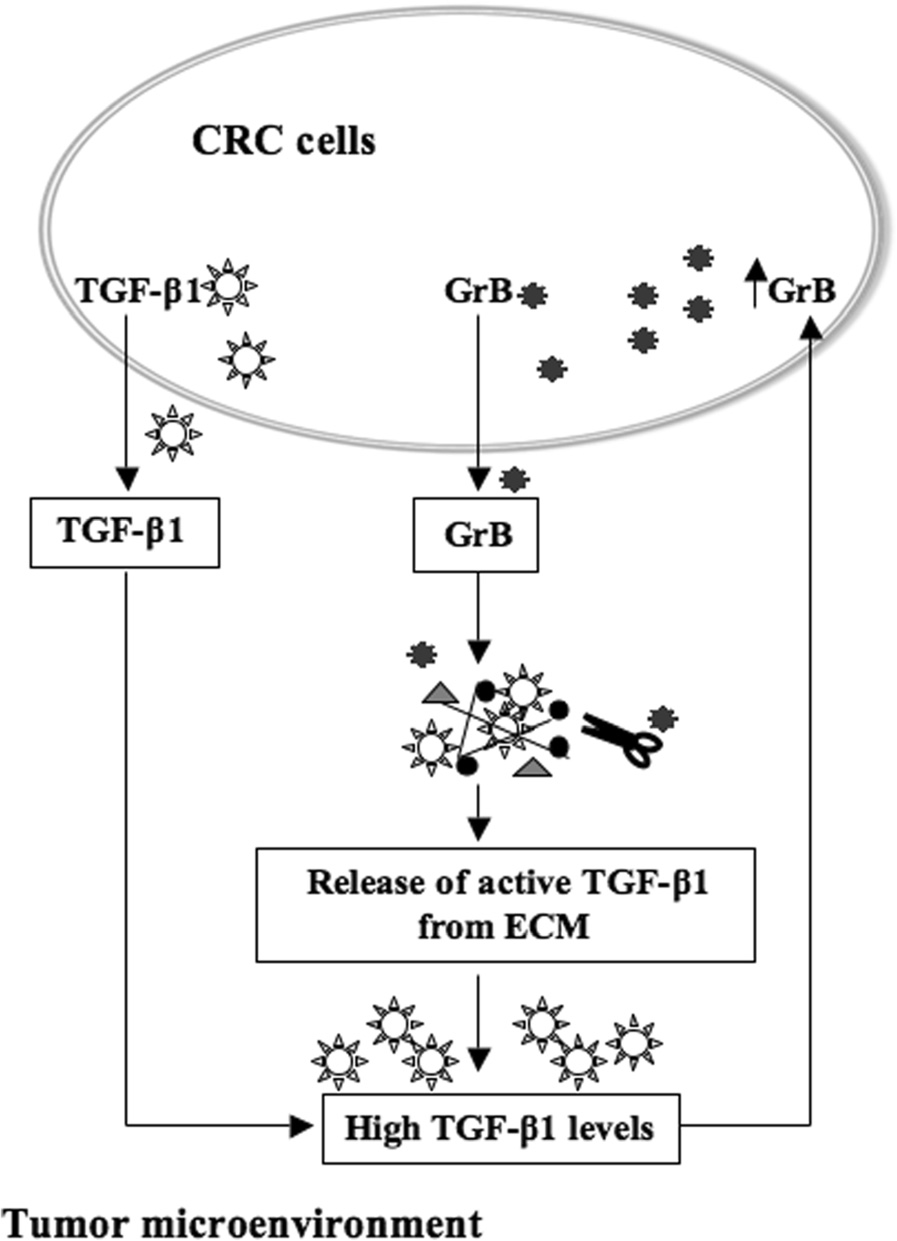
A proposed model to illustrate the reciprocal positive feedback loop between GrB and TGF-β1 in CRC cells. Cancer cells express and secrete both TGF-β1 and GrB. Extracellular GrB induces the release of active TGF-β1 by ECM (Ref. 56), leading to increase TGF-β1 levels in the tumor microenvironment. High TGF-β1 levels upmodulate tumor production of GrB (Refs. 52, 55), which further increases the extracellular level of TGF-β1

GrB function in EMT was further supported by the results derived from TGF-β1-driven EMT in CRC cell lines. We found that TGF-β1 enhanced GrB expression while inducing EMT in CRC cells. Then, of interest, GrB depletion in TGF-β1-driven EMT resulted in the inhibition of EMT induced by TGF-β1 in HT-29 cells. These data indicate a contribution of GrB in the induction of TGF-β1-driven EMT. Moreover, our finding, showing the enhancement of GrB expression by TGF-β1 in neoplastic cells, is a new and interesting data. In fact, TGF-β1 is known to suppress tumor immune surveillance by different ways, including the inhibition of anti-tumor lymphocytes-mediated cytotoxicity by the suppression of the expression of several cytotoxic effector molecules among which GrB [52]. The fact that TGF-β1 inhibits GrB expression in normal cells [52] whereas enhances GrB expression in neoplastic cells (Fig. 4b) might fit with the phenomenon denominated “TGF-β paradox”, which consists in the bi-functional effects of TGF-β in normal *vs* tumor cells [53–55]. Although the mechanism underlying this paradox still remains unknown, Zhang et al. [55] have recently proposed a deregulation of TGF-β signaling in tumor *vs* normal cells, caused by the high extracellular TGF-β level, which might be regulated by a positive feedback loop in cancer cells *vs* a negative feedback loop in normal cells. In line with this hypothesis, we can propose a reciprocal positive feedback loop between GrB and TGF-β1 in CRC cells, leading to high TGF-β1 levels in the tumor microenvironment. We assume that cancer cells not only display autocrine TGF-β production but also express and secrete GrB, which, as recently shown [56], induces the release of active TGF-β1 sequestrated by proteoglycans in ECM, leading to increase extracellular TGF-β1 levels. Thus, high TGF-β1 levels upmodulate tumor production of GrB, which further increases TGF-β1 levels in the tumor microenvironment (Fig. 7).

We have previously proposed that GrB expressed in bladder and pancreatic cancer cells promotes their invasion [18, 25]. Here, we showed that GrB depletion in HCT 116 cells significantly inhibited cell invasion through Matrigel, indicating that GrB also promotes CRC cell invasion, contributing to the CRC invasive phenotype. Consistent with these results, we found that GrB depletion in TGF-β1-driven EMT inhibited the invasion of HT-29 cells, indicating a contribution of GrB also in the promotion of invasion induced by EMT-driven TGF-β1. On the other hand, we found that constitutive expression of GrB in CRC cells was not always associated with their invasive capability, in that GrB was expressed at comparable levels in both invasive and lowly invasive CRC cells. Compatible with these results, we also found that ectopic GrB expression in CRC cells was not always associated with the enhancement of their invasion, in that invasion was upmodulated in LoVo and HT-29 cells, but not in SW480 and SW620. These results suggest that the promotion of invasion by GrB might depend on the cellular context and that a CRC subtype might exist in which additional factors might interfere with the upmodulation of invasion by GrB. To date, not much is known about the intracellular and the extracellular pathways, other than the apoptotic pathways, activated by GrB in normal and neoplastic cells. Research is needed to identify GrB targets involved in the mechanisms underlying the modulation of EMT and invasion by tumor-expressed GrB.

Lastly, here we observed that DHA, at a concentration achievable in vivo and that does not affect cell proliferation [25], is capable of inhibiting GrB expression and cell invasion in CRC cells. In addition, we showed that DHA inhibited EMT in CRC cells. These findings suggest that the inhibition of tumor-expressed GrB, EMT and invasion by DHA might represent at least some of the mechanisms underlying the anticancer activity of DHA.

## Conclusions

We reported that tumor-associated GrB contributes to tumor cell EMT and invasion, uncovering a novel potential noncytotoxic role for GrB as upmodulator of EMT. This finding might open the door for a new research on GrB function in tumors, contributing to a better understanding of the mechanisms underlying tumor metastasis and drug resistance, as well as enabling the emergence of new therapeutic opportunities. To this regard, this study showed that DHA has the capability of inhibiting CRC cell-associated GrB expression, EMT and invasion, supporting the use of DHA, a cheap dietary compound without toxic effects, as adjuvant treatment in cancer therapies.

## Abbreviations

CRC: colorectal cancer
CSCs: cancer stem cells
DHA: docosahexaenoic acid
ECM: extracellular matrix
EMT: epithelial-to-mesenchymal transition
GrB: granzyme B
TGF-β: transforming growth factor-β
WB: western blotting
